# Unveiling Salt-Wasting Congenital Adrenal Hyperplasia in an Infant: A Diagnostic Challenge

**DOI:** 10.7759/cureus.103140

**Published:** 2026-02-07

**Authors:** Satyanarayana Kummari, Mutchakarla Krishna Sravya, Mahipal R

**Affiliations:** 1 Radiodiagnosis, All India Institute of Medical Sciences, Hyderabad, IND; 2 Obstetrics and Gynecology, Government Medical College, Bhongiri, IND; 3 Radiodiagnosis, MNR Medical College and Hospital, Sangareddy, IND

**Keywords:** 21-hydroxylase deficiency, ambiguous external genitalia, autosomal recessive inheritance, clitoral enlargement, congenital adrenal hyperplasia (cah), cyp21a2 gene mutation, mineralocorticoids, non-classical congenital adrenal hyperplasia, salt-wasting cah, simple virilizing congenital adrenal hyperplasia

## Abstract

Congenital adrenal hyperplasia (CAH) is a monogenic genetic disorder with autosomal recessive inheritance. CAH can be classified into three distinct types: salt-wasting, simple-virilizing, and non-classical. In terms of severity, the salt-wasting type is the most severe form of CAH. The identification of simple-virilizing and non-classical types is challenging due to the absence of salt-wasting symptoms that require hospitalization. In this report, we describe a two-month-old female infant who was admitted to the emergency room with a history of lethargy, vomiting, and diarrhea. During the physical examination, the patient was found to have dry mucous membranes, generalized pallor, hyperpigmentation of the external genitalia, clitoral enlargement, and ambiguous genitalia. Laboratory investigations revealed the following results: 17-OH-progesterone levels at 109.19 ng/mL (N:<1.70 ng/mL), testosterone levels at 11.18 ng/dL, morning serum cortisol at 1.7 μg/dL (N:3.7-19.4 μg/dL), hyponatremia (111 mmol/L), hyperkalemia (6.0 mmol/L), and hypochloremia (85 mmol/L). The abdominal ultrasound revealed hyperplasia of the bilateral adrenal glands (right>left), normal uterus and ovaries, and absence of bilateral testicles. A mutation in the CYP21A2 gene was established by genetic testing. A diagnosis of the salt-wasting type of CAH was made. After 11 days of treatment, the patient had improved and was discharged. At the age of 12 months, the mother observed morning erections and further clitoral enlargement. The administration of fludrocortisone and oral hydrocortisone resulted in substantial improvement. This case report aims to describe a rare instance of salt-wasting CAH and highlight the challenges related to the early identification and management of ambiguous genitalia, enabling prompt intervention to prevent irreversible outcomes.

## Introduction

Congenital adrenal hyperplasia (CAH) is a monogenic genetic disorder with autosomal recessive inheritance [1]. The three main categories of CAH are salt-wasting, simple-virilizing, and nonclassical versions. The classical form encompasses simple-virilizing and salt-wasting types. The worldwide prevalence of classical CAH is between one in 13,000 and one in 15,000 live births. Seventy-five percent of patients exhibit salt-wasting, whereas the remainder present with simple-virilizing [2]. Deficiencies in several enzymes lead to CAH, inhibiting the conversion of cholesterol to cortisol. The majority of CAH cases, over 90%, are caused by an absence of 21-hydroxylase (21-OH). Clinical signs and symptoms exhibit significant variability based on the concentrations of glucocorticoids, mineralocorticoids, and sex hormones [3].

The most severe type of CAH is commonly referred to as salt-wasting adrenal hyperplasia. It is challenging to identify the nonclassical and simple-virilizing types since they do not exhibit salt-wasting symptoms that require hospitalization [4]. Newborn screening programs have been implemented to identify CAH at an early stage, mitigate diagnostic delays, and avert severe salt-loss episodes. However, newborn screening for CAH is not obligatory in India [5,6]. In this report, we describe a two-month-old female infant with the salt-wasting type of CAH.

## Case presentation

**Figure 1:**
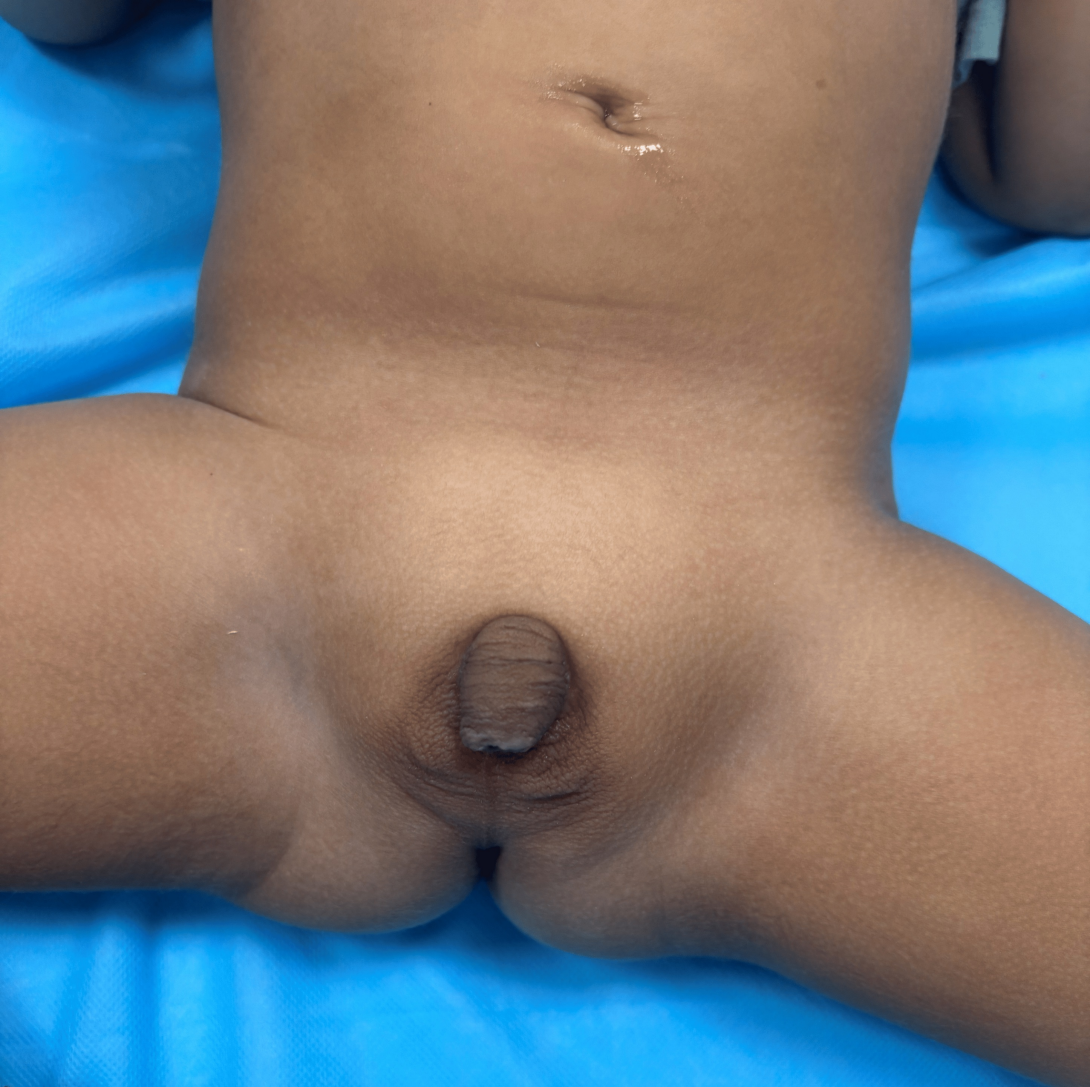
Genital examination showing pigmented, fused, and symmetrical genital folds with rugae and clitomegaly with a phallic appearance

A two-month-old female infant was admitted to the emergency department due to significant dehydration brought on by lethargy, five bouts of vomiting, and 10 episodes of diarrhea over 24 hours. During the physical examination, the patient was found to have dry mucous membranes, a marginally depressed fontanelle, generalized pallor, and a blood pressure reading of 80/50 mmHg. There was evidence of hyperpigmentation of the external genitalia, clitoral enlargement, and ambiguous genitalia (Figure 1).

Laboratory investigations revealed the following results (Table 1).

**Table 1:** Laboratory findings in a two-month-old female infant with suspected salt-wasting CAH CAH, congenital adrenal hyperplasia

Test parameter	Measured value	Reference range
17-OH-progesterone	109.19	<1.70 ng/mL
Testosterone	11.18	<5-20 ng/dL
Morning serum cortisol	1.7	3.7-19.4 μg/dL
Sodium (Na)	111	135-145 mmol/L
Potassium (K)	6.0	3.5-5.0 mmol/L
Chloride (Cl)	85	96-106 mmol/L

**Figure 2:**
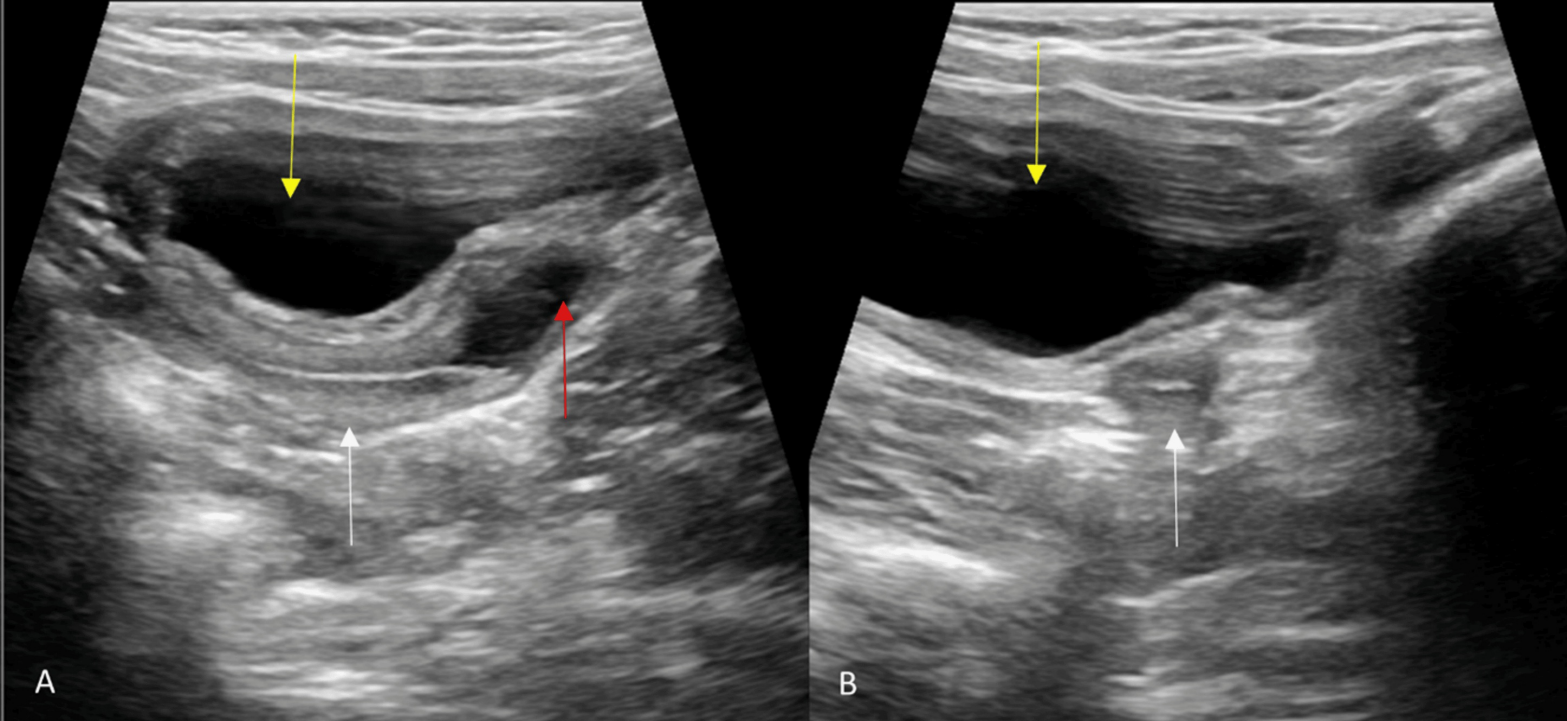
Pelvic ultrasonography images: A. Sagittal image shows the uterus (white arrow), mild fluid in the vagina (red arrow), and urinary bladder (yellow arrow); B. Axial image shows the uterus (white arrow) and urinary bladder (yellow arrow)

The abdominal ultrasound revealed hyperplasia of the bilateral adrenal glands (right>left), a normal uterus, cervix, vagina, and both ovaries, as well as an absence of bilateral testicles. The uterus, cervix, and both ovaries were normal in size, shape, echogenicity, and location. There was a fluid collection in the vagina (hydrocolpos). No other significant abnormalities were detected in the abdomen and pelvis (Figure 2).

Hormonal analysis and karyotyping were performed. Genetic testing identified a mutation in the CYP21A2 gene. A diagnosis of the salt-wasting type of CAH was established. To begin treatment immediately, oral prednisone at a dosage of one mg/day was administered along with sodium chloride supplementation. Following 11 days of treatment, the patient showed improvement and was discharged with a regimen of 2.5 mg/day of hydrocortisone and salt supplements.

The mother reported that the infant had significant clitoral enlargement and morning erections by the time the child was one year old. Meanwhile, the infant weighed only 4.6 kg and measured 63 cm in height. Eventually, the family was prescribed fludrocortisone and oral hydrocortisone, which resulted in significant improvements in the child's health.

## Discussion

CAH is a monogenic genetic disorder with autosomal recessive inheritance [1]. The three main categories of CAH are salt-wasting, simple-virilizing, and nonclassical versions. The incidence of nonclassical CAH is significantly higher. The classical form encompasses simple-virilizing and salt-wasting types [2]. Deficiencies in several enzymes lead to CAH, inhibiting the conversion of cholesterol to cortisol. The majority of CAH cases, over 90%, are attributable to a deficiency of 21-OH, as seen in the current case. The current case is a salt-wasting type of CAH (Table 1). Clinical signs and symptoms exhibit significant variability based on the concentrations of glucocorticoids, mineralocorticoids, and sex hormones (Table 2) [3].

**Table 2:** Clinical manifestations and hormonal features of CAH CAH, congenital adrenal hyperplasia; ACTH, adrenocorticotropic hormone

Manifestation	Salt-wasting		Simple virilizing		Non-classic	
	Male	Female	Male	Female	Male	Female
Age at diagnosis	Newborn - 6 months	Newborn - 1 month	2 - 4 years	Birth - 2 years	Childhood	Adolescence/adulthood
External genitalia	Normal	Ambiguous	Normal	Ambiguous	Normal	Normal or clitoromegaly
Aldosterone	Reduced	Normal	Normal	Normal	Normal	Normal
Renin	Elevated	Normal/increased	Normal	Normal	Normal	Normal
Cortisol	Decreased	Decreased	Normal	Normal	Normal	Normal
17-hydroxyprogesterone	>20,000 ng/dL	10,000-20,000 ng/dL	1,500-10,000 ng/dL, after ACTH stimulation	1,500–10,000 ng/dL - after ACTH stimulation	Mildly elevated	Mildly elevated
21-hydroxylase activity	Absent	1-10%	10-75%	10-75%	20-60%	20-60%

The most severe variant of CAH is the salt-wasting type. When enzyme activity is completely absent, cortisol and aldosterone levels are significantly lowered, while androgen levels rise concurrently, causing females to become virilized during pregnancy. In the absence of early care, newborns may encounter life-threatening salt-loss crises within weeks after birth. It is challenging to identify the simple-virilizing type because it does not exhibit salt-wasting characteristics that require admission; instead, it exhibits ambiguous genitalia from birth, with virilizing characteristics progressively emerging [4].

The condition is caused by a deficiency of enzymes, specifically 21-hydroxyprogesterone (OHP), which is essential for the synthesis of cortisol. As a result of the low level of cortisol, the hypothalamus-pituitary axis responds by increasing the secretion of adrenocorticotropic hormone (ACTH). This is responsible for the hyperplasia of the adrenal cortex and the mass production of the precursor to cortisol, which results in unique clinical signs and symptoms [7]. Therefore, the diagnosis of this condition is determined by the elevation of 17-OHP (non-diagnostic if it is <200 ng/dL, diagnostic if it is >800 ng/dL, and intermediate values necessitate an ACTH stimulation test), as well as progesterone metabolites and the precursor to cortisol, dehydroepiandrosterone sulfate. The treatment of choice for CAH is hormone replacement [8].

The treatment for both simple-virilizing and salt-wasting types in children is the same. A daily dose of 12-18 mg/m² of glucocorticoids, primarily hydrocortisone, is administered to support healthy growth and development. It is also possible to start treatment with mineralocorticoid (fludrocortisone) at doses of 0.05-0.2 mg per day [6]. The management of CAH in adult patients is still debatable [9-11]. Following the completion of epiphyseal closure, it is recommended that patients with salt-wasting and simple-virilizing types receive treatment with long-acting glucocorticoids, such as dexamethasone, prednisolone, and other similar medications. The recommended nighttime dose of these medications ranges from 0.25 mg to 0.75 mg; in addition, hydrocortisone can be administered in either a single or several doses. Nevertheless, patients with elevated plasma renin activity and aldosterone levels are the only ones prescribed mineralocorticoids (fludrocortisone), which aid in regulating 17-OHP levels [6].

Since then, newborn screening programs have been implemented to diagnose CAH at an earlier stage, reducing diagnostic delays and taking preventive measures against severe episodes of salt loss. Additionally, because undetected male cases were historically more common and frequently fatal, this screening has eliminated gender-based discrepancies in diagnosis. Due to their cultural surroundings, non-fatal, simple-virilizing CAH cases remained misdiagnosed and were raised as men prior to the required newborn screening [5]. Nonetheless, newborn CAH screening is not obligatory in India. Recent developments in genetic analysis have made it possible to identify a large number of CAH variations, which has improved diagnostic accuracy even more [6].

## Conclusions

CAH exhibits a broad range of clinical symptoms, frequently without a direct association between genotype and phenotype. This case highlights the significance of maintaining a high level of clinical suspicion, particularly when dealing with cases of ambiguous genitalia. The difficulties in diagnosing and managing ambiguous genitalia are highlighted in this case study to ensure early treatment and prevent irreversible changes.

Based on our experience with this case and evidence from existing literature, expansion of newborn screening programs for CAH in India may be beneficial for facilitating earlier detection, reducing diagnostic delays, and potentially minimizing the risk of severe salt-loss episodes and delayed management. However, the feasibility, cost-effectiveness, and broader public health implications of universal screening require further systematic evaluation in the Indian context.

## References

[1] Lajic S, Nordenström A, Ritzén EM, Wedell A (2004). Prenatal treatment of congenital adrenal hyperplasia. Eur J Endocrinol.

[2] Yau M, Gujral J, New MI (2000). Congenital Adrenal Hyperplasia: Diagnosis and Emergency Treatment. Endotext [Internet].

[3] El-Maouche D, Arlt W, Merke DP (2017). Congenital adrenal hyperplasia. Lancet.

[4] Cho SY, Ko JM, Lee KA (2016). A diagnostic algorithm after newborn screening for 21-hydroxylase deficiency. J Korean Soc Inherit Metab Dis.

[5] Khattab A, Yau M, Qamar A (2017). Long term outcomes in 46, XX adult patients with congenital adrenal hyperplasia reared as males. J Steroid Biochem Mol Biol.

[6] Fritz MA, Speroff L (2026). Clinical Gynecologic Endocrinology and Infertility. https://obgyn.lwwhealthlibrary.com/book.aspx?bookid=1227&sectionid=0.

[7] Miller WL, Levine LS (1987). Molecular and clinical advances in congenital adrenal hyperplasia. J Pediatr.

[8] Bongiovanni AM, Root AW (1963). The adrenogenital syndrome. N Engl J Med.

[9] Merke DP (2008). Approach to the adult with congenital adrenal hyperplasia due to 21-hydroxylase deficiency. J Clin Endocrinol Metab.

[10] Antal Z, Zhou P (2009). Congenital adrenal hyperplasia: diagnosis, evaluation, and management. Pediatr Rev.

[11] Lee PA, Houk CP (2010). Review of outcome information in 46, XX patients with congenital adrenal hyperplasia assigned/reared male: what does it say about gender assignment?. Int J Pediatr Endocrinol.

